# Interesting Case of Aneurism near the Origin of the Aorta

**Published:** 1844-05-18

**Authors:** 


### THE MEDICAL EXAMINER,

### And Record of Medical Science.

Vol. VII.] PHILADELPHIA, SATURDAY, MAY 18, 1844. [No. 10.

### INTERESTING CASE OF ANEURISM NEAR THE ORIGIN OF THE AORTA.

*Philadelphia*, 109 *S. Tenth st.*, *May* 14*th*, 1844.

My Dear Sir:—The subject of the following case, which has been well drawn up by Drs. Burwell and Carey, at the time resident physicians of the Philadelphia Hospital, was presented by me, on more than one occasion, to the clinical class during the last session, and excited great interest and attention, not only among the members of the class but among the different physicians attached to the Institution, and the medical gentlemen who visited it. The remarks which I made to the clinical class have been given in epitome in the Examiner, for Feb. 10, and for April 6, 1844. Accurate and creditable as these reports are to the gentlemen who prepared them, they are necessarily, however, imperfect. An interesting point in the case was the presentation of the tumour on the left side of the sternum, and the consequent difficulty in discriminating as to its source, whether aortic or cardiac. It was even doubted at one time, by intelligent observers, whether the fluid contained in the tumour were not pus, and whether the heaving expansive movement might not be owing to the pulsations of the heart being communicated to a purulent collection seated anterior to that viscus. Little doubt, however, could exist in regard to the aneurismal character of the tumour; but the difficulty was to decide as to its origin: whether it were a partial aneurism of the heart, or an aneurism originating at the very commencement of the aorta and passing in front of the heart. The result shewed that it was the latter; and the very position of the aneurismal tumour, situate wholly on the anterior or right heart, sufficiently explained the difficulty of diagnosticating between the two.

In many cases of aneurism of this nature, the rupture of the parietes takes place either into the pericardium, or into the right heart. In this case, the cavity of the pericardium was obliterated by the passage of the tumour along the anterior heart, and by the consequent adhesions; whilst around the posterior it was universally and closely adherent. Where the aneurism came in contact with the parietes of the right ventricle, these were only a line in thickness, so that it is somewhat singular, that the aneurism did not burst into that cavity, under all the difficulties in the absorption of soft parts, cartilages, and bones, which interfered with its progress towards the surface. The pressure of the aneurism on the right ventricle, and the consequent impediment to its action, may account for the hypertrophy of other portions of the parietes of that cavity.

The harsh and loud sound—louder, it seemed to me, than I had ever heard accompanying the second sound of the heart—was the cause of much embarrassment. This sound was heard most strongly over the tumour,—decidedly more so than over the semilunar valves, the closure of which, by the refluent blood, in the diastole of the ventricles, is generally presumed to be the cause of the normal second sound of the heart. The fact of the greater intensity of sound over the tumour induced me to suggest to the class, that the aneurism might proceed from immediately above the semilunar valves, so that the sound of those valves in action was conveyed immediately to the ear through the aneurismal tumour. I am, however, in great doubt, not only from this case, but from many others which I have witnessed, whether many of these loud sounds accompanying the dilatation of the heart are really seated in the semilunar valves. In this case the valves were not materially altered, and although they might have admitted of some degree of regurgitation, it could scarcely have produced so loud a sound as was heard during the diastole. Moreover, both sounds of the heart could be distinguished on auscultating the dorsal region of the thorax; and the loud sound accompanying the second sound was probably caused, as in other cases of chronic endo-aortitis, by the blood in the aorta reflowing towards the heart during the diastole, and passing over the lining membrane roughened by ossific and other depositions. It could scarcely be caused by the refluent blood from the aneurismal tumour in its passage into the aorta.

The day before the man died,—becoming weary of existence, and believing until the last, that the tumour was one from which he might be relieved by an opening made into it,—he passed in a pin, up to the head, and moved it about in all directions. Owing to the copious depositions of fibrin near the surface, this operation was not followed by any hemorrhage: sometime afterwards, however, as will be seen from the report, several ounces of blood were lost.

I am, my dear Sir, very truly yours,

Robley Dunglison.

Professor R. M. Huston.

—

[Reported by Drs. Burwell and Carey, Resident Physicians.]

John Kissal, aged 50 years, born in Philadelphia, and a sailor by occupation, entered the Medical Wards in December, 1843, complaining of disease of the heart. He is about 5 feet 4 inches high, with brown hair and light-blue eyes; emaciated; his countenance is expressive of the greatest anxiety; face and lips moderately livid, and with some œdema of lower eyelids. He is obliged to be kept raised in bed all the time, and is unable to assume the recumbent position, or to lie on either side.

He says he has been a healthy, hearty man, with the exception of having the ague, until about five years ago, when he was confined to his bed from six weeks to two months with acute articular rheumatism, affecting nearly every joint in his body. It was nearly a year after this before he could get out to work. He cannot say that at this time he felt any particular uneasiness about the heart. He does not recollect that he was short-breathed before the attack of rheumatism; but afterwards he remembers distinctly of being short-winded on running, and when engaged in fishing. With this exception he enjoyed excellent health until eighteen months ago, when he experienced some pain in the right side, which afterwards shifted to the left side with increased difficulty of breathing, and for which he came to the Hospital, expecting soon to be cured.

A diagnosis of valvular disease with cardiac dropsy was then made. He remained in the wards all winter, and was sent out in May last to another ward, where he has since been. He now complains, —1st. Of constant and severe pain in the region of the spine between the shoulder blades, which constitutes his greatest suffering. The parietes of the chest anteriorly, in the region of the heart, are very tender on percussion.

2dly. Of dysphagia, sometimes confined to that portion of the œsophagus opposite the heart, and at other times experienced all through the tube.

3dly. The respiration varies much. Some days it is only thirty-two in a minute; on others it is as high as sixty, and then exactly synchronous with the pulse. It is generally short, with inability to hold the breath long.

4thly. The action of the heart is regular and labouring, and the spiral movement of systole is well shown, being from left to right.

5thly. The impulsion corresponding with the action of the heart is heaving and strong in a marked degree. The most marked impulsion is perceived in a small tumour, which formed during the last month between the 3d and 5th ribs on the left side and just at the margin of the sternum. The cartilage of the fourth rib seems to be pushed aside or partially absorbed; the sternum is tilted up by it. This tumour is accompanied with great prominence of the præcordial region, all of which is seen to heave with every impulsion of the heart. There seems to be a withdrawal of the apex of the heart from the region below the nipple, at every beat, with a very sensible retraction or drawing in of this part of the chest; but upon applying the fingers there, the beat can be felt during the systole.

6thly. The pulse beats from 64 to 80 in a minute. It is hard and tensely prolonged except when the patient is under some excitement, when it becomes softer and smaller. There is no pulsation in the veins of the neck, nor are they at all prominent: neither abnormal pulsation nor thrill can be felt behind the top of the sternum nor in the carotids and subclavians.

7thly. Percussion is resonant throughout the right lung anteriorly, and under the clavicle of the left side; but it is perfectly flat in most of the space between the third rib left side, and the base of the chest, and from the middle of the sternum to near the left nipple.

8thly. There is a very well marked thrill felt with the diastole of the heart in the pulsating tumour and to the left of it for two inches. It is less marked under the left clavicle and upper half of the sternum and at the apex of the heart. There is little or nothing of this during the systole.

9thly. The first sound is confused, short, and rumbling, and much confounded with the impulsion. It can be heard loudest over the tumour: over the sternum between the 2d and 3d ribs of both sides it has a slight bellows character as heard through the stethoscope in addition to the rumbling sound.

10thly. The second sound is a hoarse, distinct rasp, and prolonged throughout the interval. It is distant, and on a low key, like whispered *hoo*. It can be heard loudest over the tumour, then over the sternum between the third and fourth ribs, making with the *first* sound, a saw sound in this region. It is also heard over the lower portion of the sternum, and in the region of the apex of the heart, but fainter than above the tumour. It is accompanied everywhere with the thrill before spoken of.

Both sounds of the heart can be heard posteriorly between the scapulæ, rather louder on the left side than on the right—the first sound clear, and the second roughened. They both sound distant and feeble in this place.

11thly. He has considerable anasarca of the lower limbs, which varies much in amount at different times.

February 27th.—The patient's condition remains much the same. The tumour is gradually enlarging, and has now become about two inches in diameter at the base; it is acuminated, and its end is becoming a little red and violet coloured. The impulse in the tumour is expansive from the centre, and extends to the parietes of the chest about it. The sounds of the heart above the tumour are as before, but less loud near the apex. In other respects, the man is as comfortable as could be expected. His appetite is good and bowels regular.

The pain in the back, the tenderness of the chest, the anxiety, and blueness of countenance, and hurried respiration all remain the same. He is comfortable only when under the use of laudanum.

March 16th.—The respiration and pulse each amount this morning to 84 in the minute. The beats of the heart are regular and of good force. The tumour has enlarged rapidly the last four or five days. Its lower surface is abrupt, whilst the upper and that to the left side are shelving off towards the left axilla, raising very sensibly the parietes of the chest. The pain is most severe, as it has always been, in the back, about the 6th, 7th, and 8th dorsal vertebræ, which are tender to pressure. The chest is very sensible to the air, and its exposure for some minutes causes him more pain than moderate pressure with the fingers. The lower limbs are again becoming quite anasarcous; urine decidedly albuminous.

April 8th.—The tumour has now assumed an elliptical form, extending from an inch and a half to the left of the nipple to the right side of the sternum, and from the 3d to the 6th rib. It is most acuminated over the junction of the fifth rib with the sternum, and begins here to look black. A second tumour is apparent to the right of this, near the right margin of the sternum, in which the impulsion is felt as in the first tumour, it being in fact but a part of this.

The man's countenance appears decidedly worse than a week ago. It has become anasarcous. The anasarca has affected the scrotum, and increased correspondingly in the legs and thighs. Abdomen not examined.

April 12th.—Anasarca general, abdomen tense, slight oozing of blood from the livid spot in the centre of the tumour.

April 20th.—The patient's sufferings seem to have increased since last date. Pulse 64. Respiration 30. The tumour now measures twelve inches laterally, extending from the nipple on the right side to two inches beyond the nipple on the left; eight inches from one inch below the top of the sternum to the ensiform cartilage; and five inches from the apex to the level of the sternum. The livid spot at the apex is three inches in diameter, and its margin well defined.

April 22d.—The patient says he accidently stuck a pin into the tumour, but after it had entered as far as the head, he moved it about to enlarge the aperture. Upon withdrawing the pin, blood did not follow immediately.

12, P. M.—There has been a gush of blood (f.℥viij.) which was checked by slight pressure.

April 23d.—He expired at one o'clock this morning, with comparative ease, and with no further hemorrhage.

*Necroscopy thirty-three hours after death.*—The tumour has subsided in some degree. Much infiltration of the lower extremities. Dulness on percussion at the base of tumour, but extending no great distance from it. Commencing at the sternal extremities of the clavicles, incisions were made through the integuments along the junction of the ribs with their cartilages. To obtain a view of the tumour *in sitû* it was found necessary to cut off three or four inches of the ribs. Some effusion existed in the right pleura; the right lung was healthy. Slight effusion in the left pleura, and general adhesion between the pleura costalis and pleura pulmonalis, with the exception of the upper lobes. The left lung was adherent to the pericardium; and compressed and consolidated at its posterior and lower portion. All the large vessels being tied some distance from their origin, were cut off beyond the ligatures; the tumour with the heart was removed; the pericardium was universally adherent to the heart; the measurement of the heart, from the base to the apex was 6 inches. The walls of the right ventricle were half an inch thick, except where the aneurism came in contact with them, and there they were only a line in thickness, exclusive, of course, of the columnæ carneæ, which seemed enlarged. The walls of the left ventricle measured three quarters of an inch. The circumference of the mitral valves was five and a quarter inches. That of the tricuspid five inches. Septum between the ventricles half an inch. The lining membrane of the heart presented a pale appearance. Slight adventitious deposits with appearance of recent inflammation upon the corpora Arantii. One of the semilunar valves of the aorta was slightly bound down, measuring a line less at its base than the others. These valves were, however, comparatively healthy. Numerous ossific deposits along the aorta. From just beyond the sinus of Valsalva in the aorta, an aperture an inch in diameter presented itself: this was the opening of an aneurismal sac, which involved the coronary artery, and passed beneath the pericardium over the right side of the heart. It was a true aneurism, extending along the heart and intimately connected with it until it came opposite the 4th, 5th, and 6th ribs at their junction. An inch of the whole sternum, and two inches on either side above, with the cartilages of the 5th, 6th, and 7th ribs, were eroded and absorbed. The sac would contain nearly a quart. The blood in the anterior portion of the sac was recently coagulated. There were laminæ of fibrin as usually seen in the interior of aneurisms.

